# Comparative Physiological and Transcriptomic Profiling Offers Insight into the Sexual Dimorphism of Hepatic Metabolism in Size-Dimorphic Spotted Scat (*Scatophagus argus*)

**DOI:** 10.3390/life11060589

**Published:** 2021-06-21

**Authors:** Huapu Chen, Dongneng Jiang, Zhiyuan Li, Yaorong Wang, Xuewei Yang, Shuangfei Li, Shuisheng Li, Wei Yang, Guangli Li

**Affiliations:** 1 Guangdong Research Center on Reproductive Control and Breeding Technology of Indigenous Valuable Fish Species, Key Laboratory of Marine Ecology and Aquaculture Environment of Zhanjiang, Fisheries College, Guangdong Ocean University, Zhanjiang 524088, China; chenhp@gdou.edu.cn (H.C.); dnjiang@gdou.edu.cn (D.J.); g_yl903@163.com (Z.L.); yaorongwang217@126.com (Y.W.); 2 College of Life Sciences and Oceanography, Shenzhen University, Shenzhen 518052, China; yangxw@szu.edu.cn (X.Y.); szu_sfli@163.com (S.L.); 3 State Key Laboratory of Biocontrol, Sun Yat-sen University, Guangzhou 510275, China; lshuish@mail.sysu.edu.cn; 4 Guangdong Province Key Laboratory for Aquatic Economic Animals, Sun Yat-sen University, Guangzhou 510275, China; 5 Food and Environmental Engineering Department, Yangjiang Polytechnic, Yangjiang 529566, China

**Keywords:** *Scatophagus argus*, sexual size dimorphism, liver, RNA-Seq, metabolism, enzyme activity

## Abstract

The spotted scat (*Scatophagus argus*) is an economically important cultured marine fish that exhibits a typical sexual size dimorphism (SSD). SSD has captivated considerable curiosity for farmed fish production; however, up till now the exact underlying mechanism remains largely unclear. As an important digestive and metabolic organ, the liver plays key roles in the regulation of fish growth. It is necessary to elucidate its significance as a downstream component of the hypothalamic-pituitary-liver axis in the formation of SSD. In this study, the liver physiological differences between the sexes were evaluated in *S. argus*, and the activity of several digestive and metabolic enzymes were affected by sex. Females had higher amylase, protease, and glucose-6-phosphate dehydrogenase activities, while males exhibited markedly higher hepatic lipase and antioxidant enzymes activities. A comparative transcriptomics was then performed to characterize the responsive genes. Illumina sequencing generated 272.6 million clean reads, which were assembled into 79,115 unigenes. A total of 259 differentially expressed genes were identified and a few growth-controlling genes such as *igf1* and *igfbp1* exhibited female-biased expression. Further analyses showed that several GO terms and pathways associated with metabolic process, particularly lipid and energy metabolisms, were significantly enriched. The male liver showed a more active mitochondrial energy metabolism, implicating an increased energy expenditure associated with reproduction. Collectively, the female-biased growth dimorphism of *S. argus* may be partially attributed to sexually dimorphic metabolism in the liver. These findings would facilitate further understanding of the nature of SSD in teleost fish.

## 1. Introduction

Sexual size dimorphism (SSD), also known as sexual growth dimorphism, is commonly defined as the significant differences in body size and growth rate between males and females [1]. As an interesting phenomenon in the animal kingdom, SSD is found to be widely encountered in teleost fish and has been described in detail in more than 20 species [2], including many farmed fish such as Nile tilapia (*Oreochromis niloticus*), yellow catfish (*Pelteobagrus fulvidraco*), and half-smooth tongue sole (*Cynoglossus semilaevis*) [3]. Unfortunately, the existence of SSD can lead to undesirable consequences (e.g., lower yield, higher feeding cost, and poorer quality) if the sex with a higher relative growth dominance does not predominate in the cultured stocks [4]. Therefore, this phenomenon has captivated considerable curiosity for farmed fish production, and subsequently great investigative efforts have been made to thoroughly comprehend the mechanisms underlying SSD, with the aim of exploring an alternative approach to improve the productivity of aquaculture.

In teleosts, somatic growth is primarily regulated by the growth hormone (GH)/insulin-like growth factor (IGF) system of the hypothalamic-pituitary-liver (HPL) axis [5]. Over the years, increasing evidence has shown the importance of interaction between the somatotropic axis and the brain-pituitary-gonad axis in the regulation of fish growth [2]. Certain physiological mechanisms underlying sexuality (for example, sex steroids) are assumed to induce a growth dimorphism during sexual and reproductive maturation [1,6]. In fish species, recent investigations focusing on the neuroendocrine growth factors have characterized the sexual expression patterns of growth hormone-releasing hormone (*ghrh*) [7,8], growth hormone (*gh*) [9,10,11,12], growth hormone receptors [13,14], and insulin-like growth factor I (*igf1*) [10,12,14]. Moreover, further understanding of the regulatory effects of sex hormones on GH/IGFs axis genes implies that these genes serve as key regulators in the occurrence of SSD [13,14,15,16,17].

Fish growth is regarded as a polygenic trait that integrates many biological processes and involves a large number of individual genes. Growth speed is also influenced by multiple physiological pathways that regulate material and energy metabolism. The gene-by-gene strategy cannot give a full knowledge of the complex regulatory processes, and even now the primary downstream signaling and biological process involved in the formation of SSD remain poorly understood. The liver acts as an important digestive and metabolic organ in the growth and development process of teleost fish and plays key roles in the regulation of nutrient utilization, metabolism of lipids, carbohydrates and proteins, endocrine, and immune homeostasis [18]. As a downstream component of the HPL axis, the liver possesses the greatest density of GH receptors [18] and in part mediates the growth-promoting effects of GH by secreting IGF-I in response to the stimulation of GH [19,20,21,22]. Given its central involvement in the regulation of growth, the liver has been widely used as a target organ to decipher the genetic and metabolic mechanisms that contribute to the significantly different growth rate of fish [18,23,24,25,26,27,28], and these findings help us to reach a better understanding of the regulation of growth. Accordingly, it is reasonable to suppose that the liver also participates in the formation process of SSD in fish species. However, up till now very little research has attempted to comprehensively evaluate sex differences in the physiological and metabolic patterns of liver tissue, which are essential to address the interaction mechanisms between liver and SSD.

The spotted scat (*Scatophagus argus*) is an increasingly important cultured marine fish with great economic potential in southeast Asia, particularly China [29]. This species has been widely cultured in Guangdong, Guangxi Province and Taiwan District with an estimated annual output value of RMB 150 Million [30]. *S. argus* is also known to exhibit a typical SSD at commercial size under either cultural or natural conditions [11,30], making it an excellent candidate model to elucidate the mechanisms of sexually dimorphic growth. Our previous studies have revealed the sex-biased expressions of *ghrh*, *gh*, and *igf1* as well as the influences of exogenous estradiol-17β (E_2_) on them [8,11,16,31], but no report on the regulatory roles of liver tissue is available and the exact physiologic and molecular mechanisms responsible for SSD is still far from clear. In this study, the differences of liver physiological indicators between male and female *S. argus* were examined and a comparative transcriptome analysis was further performed to characterize the responsive genes in the liver, aiming to provide a global view of the sexual differences in hepatic biological processes and to explore the potential pathways responsible for inducing SSD. Such data would be of value to further illuminate the nature of the mechanisms that give rise to SSD in this species.

## 2. Materials and Methods

### 2.1. Fish and Sample Preparation

In late August 2017, eggs from five dams and semen from four sires were collected by abdominal massage of mature *S. argus*. An artificial fertilization was performed and the fertilized eggs were hatched in a 2500 L tank with filtered seawater and gentle aeration. The hatched larvae were cultured in a 5 m × 5 m × 2 m pool till October 2017. The juvenile offspring were then transferred and raised in an outdoor muddy pond (12–25‰ salinity). The experimental fish were fed commercial marine fish pellets (YueHai Feeds Group Company, Zhanjiang, China) twice daily. At 14 months of age, fish were randomly sampled and anesthetized by immersing them in a 300 mg/L tricaine methanesulfonate (MS-222, Sigma, Saint Louis, MO, USA) bath. After phenotypic measurement, the anesthetized fish were dissected following blood collection from the lateral caudal vein. The determination of fish gender was performed by morphological observation of gonads. Liver and gonad tissues were collected from 20 males and 20 females, and their weights were recorded and used to calculate hepatosomatic index (HSI) and gonadosomatic index (GSI). The liver samples were quick-frozen in liquid nitrogen and then stored at −80 °C until RNA extraction and biochemical analysis. Meanwhile, small pieces of gonad and liver tissues from each fish were fixed in Bouin’s solution for histology. The blood samples were kept at 4 °C overnight, the serum was collected by centrifugation (2150× *g*, 5 min) and stored at −20 °C until use. Animal handling procedures were carried out in strict accordance with the recommendations in the Guide for the Care and Use of Laboratory Animals. The protocol was approved by the Animal Research and Ethics Committee of Guangdong Ocean University (NIH Pub. No. 85–23, revised 1996).

### 2.2. Histological Procedures for Liver and Gonad Tissues

The tissue specimens were fixed in Bouin’s solution for 20 h and then transferred into 70% ethanol, followed by dehydration in a gradient ethanol series (75–100%). The dehydrated tissues were cleared with xylene and embedded in molten paraffin, followed by serially sectioning at 6–8 μm. The sections were stained with hematoxylin and eosin (H&E), and histomorphologic evaluation was performed using a light microscope (Nikon IQ50, Tokyo, Japan). The histological developmental stages of gonads were determined according to previous description [32].

### 2.3. Measurement of Enzyme Activities and Serum Steroid Hormone Assay

The liver sample was accurately weighed and homogenized (dilution 1:10) in ice-cold 0.85% normal saline with a motor-driven tissue-cell disrupter. The homogenate was centrifuged at 900× *g* for 10 min at 4 °C, and the supernatant was used as the enzyme source. The protein content of the homogenate was measured by Folin-phenol reagent [33]. Protease activity was measured using casein as substrate as previously described [34]. The activities of amylase (AMS), lipase (LPS), glucokinase (GK), glucose-6-phosphatase (G6Pase), phosphofructokinase (PFK), citrate synthase (CS), succinate dehydrogenase (SDH), pyruvate kinase (PK), acyl-CoA oxidase (ACO), malic enzyme (ME), fatty acid synthase (FAS), glucose-6-phosphate dehydrogenase (G-6-PD), acetyl-CoA carboxylase (ACC), carnitine-acylcarnitine translocase (CACT), hepatic lipase (HL), lipoprotein lipase (LPL), superoxide dismutase (SOD), glutathione peroxidase (GPX), and catalase (CAT) in the liver were determined with commercial assay kits following the manufacturers’ instructions, and the product numbers are shown in Table S1. The estradiol-17β (E_2_) and 11-ketotestosterone (11-KT) levels in serum samples were measured by an enzyme immunoassay kit (Cayman Chemical, Ann Arbor, MI, USA), according to the manufacturer’s instruction [35]. The absorbance was measured using a photometric microplate reader (multiscan MK3, Thermo Fisher Scientific, Chelmsford, MA, USA).

### 2.4. RNA-Seq and Bioinformatics Analysis

The sex phenotype and gonad stage were determined by histological methods as previously described. A total of six liver samples (three replicates for each sex) were used for the preparation of transcriptome (RNA-Seq) sequencing libraries. The RNA-Seq process was performed as described previously [29] in accordance with the following steps: total RNA was isolated using a Trizol reagent kit (Life Technologies, Carlsbad, CA, USA), isolated RNA was quantified by a Nanodrop 2000c spectrophotometer (Thermo Scientific, Wilmington, DE, USA) and its integrity was confirmed by Agilent 2100 BioAnalyzer System (Agilent Technologies, Santa Clara, CA, USA), cDNA libraries were constructed following the Illumina RNA sequencing protocol, and sequencing of the libraries was performed using an Illumina HiSeq™ 2500 platform (Illumina, Inc., San Diego, CA, USA) that generates 125 bp paired-end (PE) reads. The sequencing data have been submitted to the Sequence Read Archive (SRA) databases of NCBI under the BioProject accession number PRJNA513082.

The bioinformatics analysis involved reads quality control, assembly, annotation, and differential expression analysis. Firstly, the raw sequencing data in fastq format were quality-controlled using SOAPnuke v1.5.0 [36]. In this step, clean data were obtained by removing adapter sequences, reads containing poly-N sequences and low-quality reads from the raw data. Then, the clean RNA-Seq data were assembled by Trinity Assembler (version: r20140717) with default parameters [37]. Functional annotation was performed by sequence alignment against public databases using BLAST 2.2.26+ software with an E-value cut-off of 1 × 10^−5^. Gene Ontology (GO) functional results were obtained by Blast2GO program [38], and GO term classification and visualization were accomplished by WEGO software [39]. The KEGG pathway annotation was analyzed by KOBAS v2.0 for pathway categories [40].

By means of expected number of fragments per kb per million reads (FPKM) method, gene expression levels were calculated using RSEM v1.2.21 [41]. DESeq2 v1.4.5 package was used to identify differentially expressed genes (DEGs), with a FDR < 0.01 and |log_2_ fold change (FC)| > 1 as the threshold for significant differential expression [42]. Up-regulated DEGs had log_2_ FC > 0 and by contrast, down-regulated DEGs had log_2_ FC < 0. GO and KEGG enrichment analyses were performed using the hyper geometric distribution test, by the phyper function in the R software package v3.4.4 (http://www.rproject.org/ (accessed on 25 April 2018)). GO term with FDR ≤ 0.05 was defined as the term of significantly enriching DEGs. A similar method was used to test the statistical enrichment of DEGs in KEGG pathways [43].

### 2.5. Real-Time Quantitative PCR (RT-qPCR) Validation

Total RNA was isolated from liver samples using TRIzol according to the manufacturer’s instructions (Invitrogen, Carlsbad, CA, USA). The RNA was then subjected to reverse transcription using a RevertAid first strand cDNA synthesis kit (Thermo Scientific, USA). RT-qPCR was performed on a LightCycler 480 system (Roche, Basel, Switzerland) using SYBR Premix Ex Taq II (TaKaRa Bio Inc., Shiga, Japan), as previously described [35]. Three independent biological triplicates and two technique repeats were performed for each sample. The reference gene β-actin was used as an internal control to normalize mRNA levels [44]. All the primer pairs are listed in Table S2. The relative gene expression levels were calculated using 2^−ΔΔCt^ method.

### 2.6. Statistical Analysis

All data were expressed as means ± standard errors (SE). Using SPSS v19.0 statistical package (SPSS Inc., Chicago, IL, USA), the two-tailed Student’s *t*-test for unpaired samples was used to statistically evaluate the differences in the mean results between groups. Statistical significance was determined at the 95% confidence level (*p* < 0.05).

## 3. Results

### 3.1. Comparisons of Growth, HSI, GSI, and Steroid Hormones Levels between the Sexes

**Figure 1:**
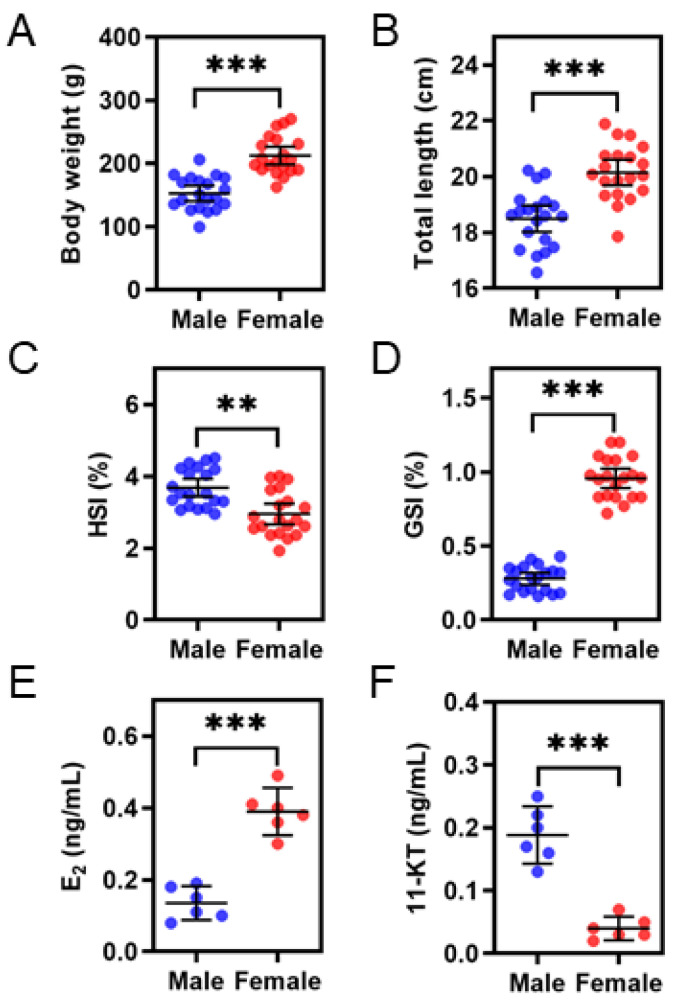
Comparisons of growth (**A**,**B**), HSI (**C**), GSI (**D**), and serum steroid hormones levels (**E**,**F**) between male and female *S. argus*. Error bars indicate the standard error of the mean (SEM) where *n* = 20 for groups (**A**–**D**) and *n* = 6 for groups (**E**,**F**). Asterisks indicate significant differences between the two groups (** *p* < 0.01, *** *p* < 0.001).

The sex of fish samples was determined by morphological observation of gonads, and further histological analysis showed that all the male and female gonads were at stage III (Figure S1). At 14 months of age, significant differences in body weight and body length were observed between male and female *S. argus* (*p* < 0.001). The mean weight of female fish (212.5 ± 30.2 g) was almost 40 percent higher than that of males (152.6 ± 26.3 g) (Figure 1A). The HSI of male fish varied from 2.95% to 4.51% and was significantly higher than that of females (*p* < 0.001) (Figure 1C). In addition, the GSI of males was 0.28 ± 0.08% and that of females reached 0.96 ± 0.14% (Figure 1D). Serum steroid hormone (E_2_, 11-KT) levels were also measured and the results differed significantly between the two sexes. Females had higher serum E_2_ levels, while males had higher serum 11-KT levels (Figure 1E,F).

### 3.2. Sex Differences in the Activity of Liver Digestive and Metabolic Enzymes

**Figure 2:**
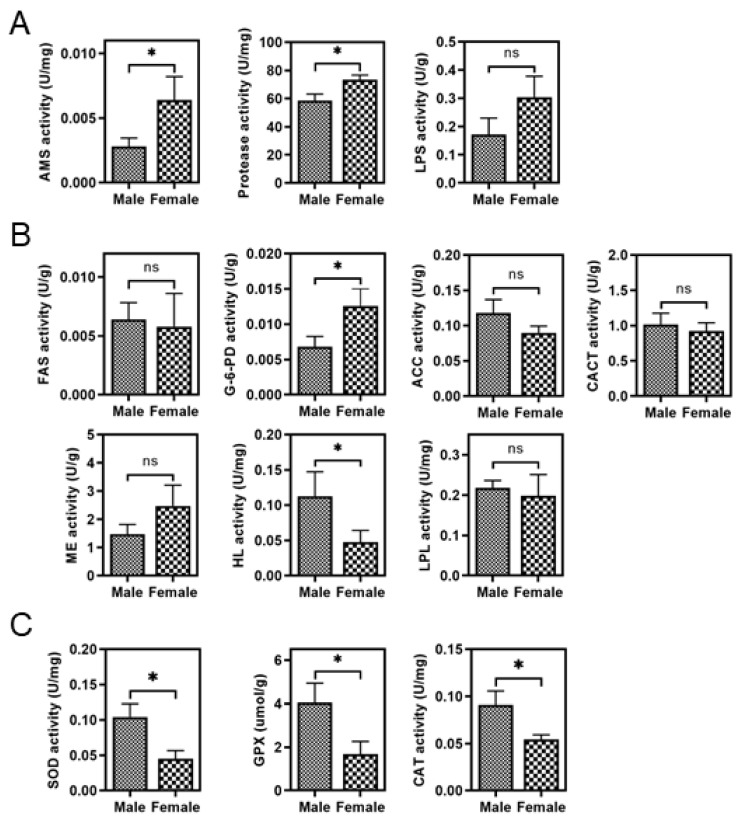
Comparative evaluation of the activity of hepatic enzymes related to digestion (**A**), lipid metabolism (**B**), and antioxidant defense (**C**) between male and female *S. argus*. Error bars indicate the SEM (*n* = 5). Asterisks indicate significant differences between the two groups (* *p* < 0.05). ns: not significant (*p* > 0.05).

The histological evaluation showed that no obvious difference in the general liver morphology was observed between male and female *S. argus* (Figure S2). Further comparison of the activity of digestive enzymes revealed that both amylase and protease activities in the liver were affected by sex, and female fish exhibited higher activities than males (*p* < 0.05) (Figure 2A). As for lipid metabolism, male *S. argus* exhibited markedly higher HL activity than female individuals, whereas female fish had higher G6-PDH activity (*p* < 0.05), the activity of other enzymes was not significantly different between the sexes (Figure 2B). Moreover, sex differences in antioxidant enzymes activities were also observed. The male group had significantly higher SOD, GPX, and CAT activities than the female group (Figure 2C).

### 3.3. RNA-Seq of the Liver Transcriptome

Illumina sequencing of the six libraries yielded 275.6 million raw reads, and the quality control resulted in 272.6 million clean reads, which is equal to 40.90 Gb sequencing data. The mean Q20 (%) and the ratio of the clean data were 94.01 and 98.93%, respectively (Table 1). *De novo* assembly generated 79,115 unigenes with a mean length of 1258 bp (Table 2). Among them, 30,339 (38.35%) unigenes were >1000 bp and 16,913 (21.38%) were >2000 bp in length (Figure S3). A total of 56,452 (71.35%) unigenes were successfully annotated in at least one of the queried databases, and 15,061 (24.28%) could be annotated in all the databases; Further analysis of the matching sequences showed that most genes (21,909; 51.83%) were related to those of *Larimichthys crocea* (Figure S4). These results reflect the high validity and reliability of assembly. To further understand the function, 22,268 unigenes were assigned into 57 2nd GO terms (Figure S5), and 37,068 unigenes were grouped into 42 2nd KEGG categories and 306 pathways (Figure S6).

### 3.4. Differential Expression Analysis

**Figure 3:**
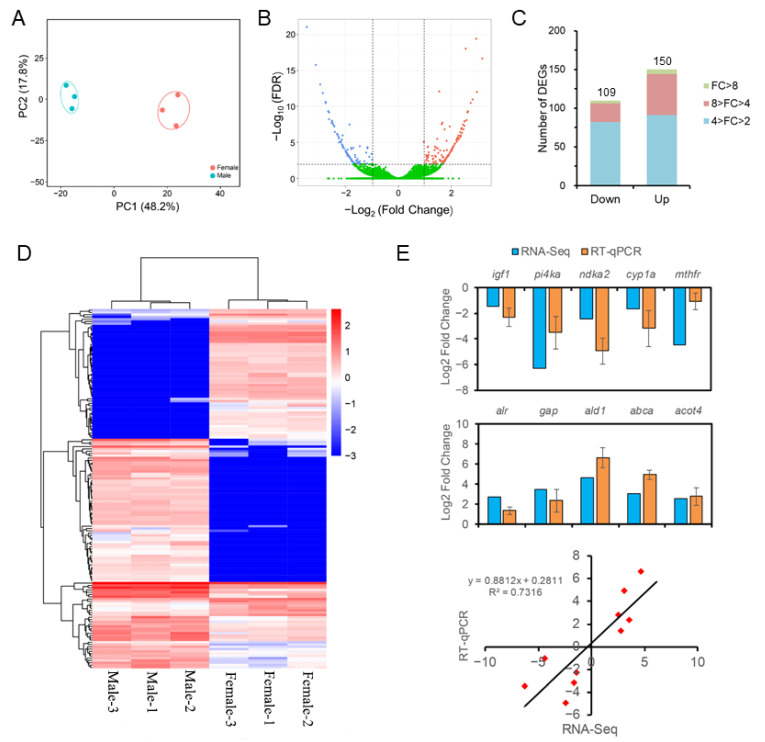
Differential gene expression between the male and female livers of *S. argus*. (**A**) Clustering of male and female samples based on the liver transcriptome profiles by principal component analysis (PCA). Three male samples (blue circles) formed a cluster and three female samples (red circles) formed another distinct cluster. (**B**) Volcano plot of the differences in gene expression. Red dots: upregulated, represent male-biased genes; green dots: downregulated, represent female-biased genes. (**C**) Distribution characteristics of DEGs with different fold-changes. (**D**) Heatmap of hierarchical cluster of DEGs for illustrating the overall pattern of gene expression among different liver samples. (**E**) Differential expression validation by RT-qPCR. Error bars indicate the SEM (*n* = 3). The consistency of log_2_ fold change between RNA-Seq data (*x*-axis) and RT-qPCR analysis (*y*-axis) is shown.

The levels of gene expression were normalized using the FPKM values and the result of principal component analysis (PCA) showed that three male samples formed a cluster and three female samples were grouped into another distinct cluster (Figure 3A), verifying that all liver samples are reliable. By comparison of the expression levels, a total of 259 genes were identified to be differentially expressed between the male and female groups. Among these DEGs, 109 genes were down-regulated and 150 genes were up-regulated in males (Figure 3B), and the slightly altered DEGs (4 > FC > 2) accounted for the largest proportion of DEGs (Figure 3C). A heatmap of hierarchical cluster of DEGs was also visualized to illustrate the overall pattern of gene expression among different sexes. Male samples were clustered into one major clade one by one and then clustered with the female clade, indicating that the livers from males and females were obviously different at transcriptional level (Figure 3D). Moreover, ten DEGs were chosen and subjected to RT-qPCR verification. The correlation analysis showed that the consistent tendencies of expression levels between the RNA-Seq and RT-qPCR results (*R*^2^ = 0.7316) confirmed the reliability and accuracy of gene expression levels quantified by transcriptomic analysis (Figure 3E).

### 3.5. The Enriched GO Terms and KEGG Pathways

**Figure 4:**
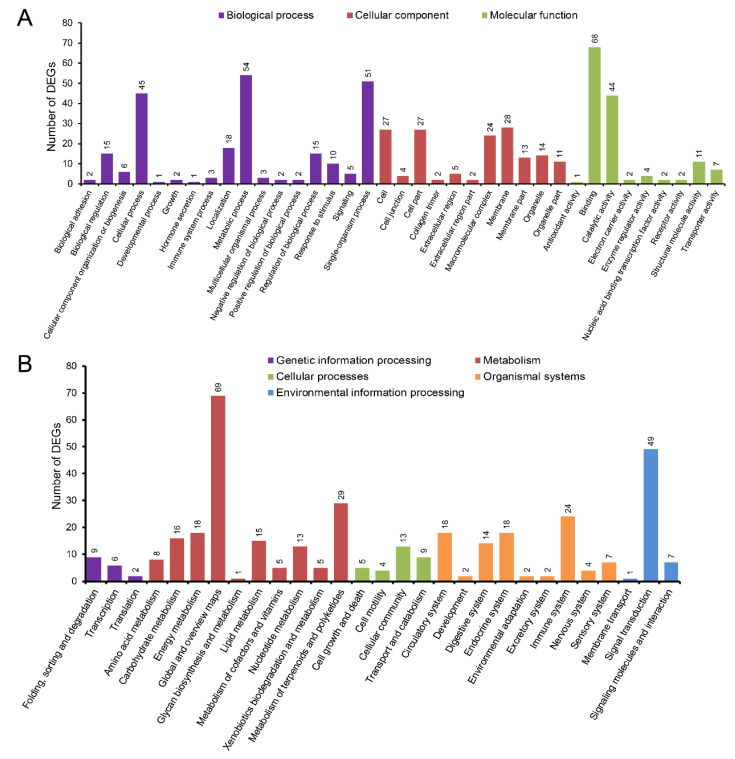
Functional annotation analyses of differentially expressed genes (DEGs) based on the categorization of GO terms (**A**) and KEGG pathways (**B**). The horizontal axis indicates the GO terms or KEGG pathways, and the vertical axis represents the number of DEGs.

A GO functional analysis was performed and the result indicated that the 259 DEGs were finally assigned to 37 2nd level GO terms. Of these functional terms, DEGs were mainly classified into the ‘binding’, ‘metabolic process’, ‘single-organism process’, ‘cellular process’, and ‘catalytic activity’ terms (Figure 4A). According to GO enrichment analysis, the top three GO terms involved in biological processes included ‘oxygen transport’ (GO:0015671), ‘ammonium transport’ (GO:0015696), and ‘actin filament severing’ (GO:0051014); the top three molecular functions terms were ‘structural molecule activity’ (GO:0005198), ‘heme binding’ (GO:0020037), and ‘calcium ion binding’ (GO:0005509); the top three cellular components terms were found to be ‘keratin filament’ (GO:0045095), ‘desmosome’ (GO:0030057), and ‘membrane’ (GO:0016020) (Table S3). Furthermore, several GO terms associated with metabolic process were significantly enriched, such as ‘proteolysis’ (GO:0006508), ‘oxidation-reduction process’ (GO:0055114), ‘carbonate dehydratase activity’ (GO:0004089), ‘lipid binding’ (GO:0008289), ‘lipid transport’ (GO:0006869), and ‘carbon utilization’ (GO:0015976) (Table S3).

**Figure 5:**
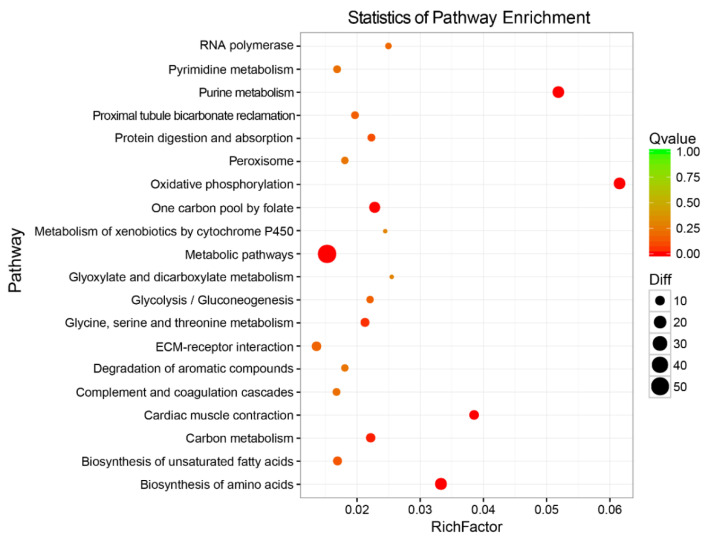
The top 20 significantly enriched KEGG pathways of differentially expressed genes (DEGs). The pathways and rich factor are shown in the vertical and the horizontal axis, respectively. The dot size indicates the number of genes and the color indicates the *q* value.

A KEGG enrichment analysis was also carried out to uncover the functional pathways defined by the identified DEGs. These DEGs were mapped to 28 2nd categories and 169 KEGG pathways (Figure 4B). Among these biological pathways, ‘global and overview maps’, ‘signal transduction’, and ‘metabolism of terpenoids and polyketides’ had the highest percentage of DEGs. Meanwhile, a total of 20 pathways were significantly enriched (*q*-value < 0.05) (Figure 5). According to their functions, these pathways, as well as the typical genes involved in them can be classified into material metabolism (carbon metabolism, lipid metabolism, and amino acid metabolism), energy metabolism (oxidative phosphorylation, ATP synthesis, and glycolysis), metabolites and ion transporters, immune system, and signal transduction (Table S4). Of which, the following KEGG pathways related to metabolism were enriched significantly: ‘biosynthesis of amino acids’, ‘biosynthesis of unsaturated fatty acids’, ‘carbon metabolism’, ‘complement and coagulation cascades’, ‘glycolysis/gluconeogenesis’, ‘glyoxylate and dicarboxylate metabolism’, ‘metabolic pathways’, and ‘oxidative phosphorylation’ (Figure 5). These functional categories would serve as a guide to reveal the differences of hepatic metabolism between the two sexes in *S. argus*.

## 4. Discussion

The growth rate is an economic trait of great interest for farmed fish and strongly affect the profitability of aquaculture. Previous reports have shown that two-year-old female *S. argus* grow about 50% larger than males [11,45]. In this study, we found that the growth performance of females was almost 40% higher than that of males, once again confirming the differential growth between the two sexes in *S. argus*. Further histological observation and ELISA assay showed that the male and female gonads were both at stage III, and male individuals had higher plasma levels of 11-KT, while females had higher levels of E_2_. In teleosts, the major androgen and estrogen essential to gonadal development and sex maintenance are 11-KT and E_2_, respectively [46]. The potential correlations of steroid hormones levels with sex differences in growth performance have been documented in fish [6,47]. In agreement with these previous reports, here we also observed a direct relationship between sex steroids and growth performance in *S. argus*. Furthermore, there are a number of examples in which treatments with sex steroids have been demonstrated to disparately affect the growth efficiency of fish [48,49,50,51]. Specifically, androgenic steroids generally stimulate growth in fish species exhibiting male-biased SSD, while estrogenic steroids mainly have growth-promoting effects on fish species showing female-biased SSD [50,51]. *S. argus* exhibits female-biased SSD, suggesting the involvement of sex hormones, particularly estrogen, in this phenomenon. In female and male fish, sex steroid hormones are present in different concentrations and have different physiological effects. Along with their regulatory roles in fish reproduction, sex steroids have also been known to modulate other important biological processes like food intake, utilization of nutrients, energy metabolism and allocation, and stress response [47,51,52,53], exerting sex-dependent effects on somatic growth and thereby leading to the occurrence of SSD. These findings provide a valuable clue to reveal the mechanisms involved in sexual growth difference.

In general, SSD is traditionally attributed to gender differences in either energy acquisition or allocation during reproduction and growth [49], both of which have been reported in teleosts [54,55]. Aquatic animals acquire nutrient and energy almost solely by feeding, digestion, and absorption. Absorption and utilization of nutrients are strongly dependent on the activity of digestive enzymes and positively contribute to the growth capacity of fish [53]. It has been demonstrated that the higher growth performance of male tilapia may be due to a greater capacity for digesting and metabolizing nutrients [56]. As a useful index for evaluating the growth performance of fish, several studies have revealed that sex has a direct effect on the activity of enzymes in digestive organs including the liver [45,53,57,58]. Compared with male adult guppy (*Poecilia reticulate*), the specific activities of amylase and total protease were relatively higher in the female population that exhibits a growth advantage, while the activity of lipase was higher in males than females [53]. Also, Wu et al. [45] reported the sexual differences in the activity of digestive tract enzymes and their potential relationship with SSD in *S. argus*. In the current study, we found that female *S. argus* had significantly higher hepatic amylase and protease activities than males; furthermore, several DEGs involved in proteolysis process such as transmembrane protease serine 2, transmembrane protease serine 9, and carboxypeptidase E were identified as female-biased genes (Table S4), suggesting a better utilization of protein in females. These results are essentially consistent with previous findings by our group in which both higher daily food consumption and food conversion efficiency were observed in females [45]. In summary, we may speculate that the faster growth rate of female *S. argus* is in partial related to the higher digestive enzyme activities that could result in a better food utilization efficiency.

It has also been known that the proximate mechanisms for SSD in fish may be associated with sexual differences in energy metabolism and allocation during reproduction and growth, for example, a higher energy investment in female reproduction compared to male tilapia [17]. Over the years, although the direct relationship between SSD and energy repartition during sexual maturation has been less studied, the potential roles of gonadal steroids in the modulation of energy allocation in fish have been especially investigated to elucidate the underlying mechanisms. It is well established that treatments with exogenous sex steroids have disparate effects on the energy allocation patterns. For example: changes in androgen levels during the reproductive season were shown to correlate with changes in energy allocation [59]; higher plasma testosterone levels in gilthead sea bream elicited hepatic metabolic changes that may be related to the energy reallocation process [52]; in *Tachysurus fulvidraco*, E_2_ administration promoted energy allocation to the liver and treatment with 17α-methyltestosterone reduced energy allocation to the ovary, suggesting that the growth suppression effect of E_2_ are induced by different energy allocation [60]. In this study, although the activity of seven representative carbohydrate metabolism-related enzymes was detected to be not significantly different between the sexes (Figure S7), a few processes involved in energy metabolism were enriched and in particular, there was a remarkable upregulation of transcripts (e.g., phosphoglycerate mutase 2, glyceraldehyde 3-phosphate dehydrogenase, NADH dehydrogenase, ATP synthase) associated with glycolysis, oxidative phosphorylation and ATP synthesis pathways (Table S4), implicating a steroid hormone-mediated sexual dimorphism in the energy use of *S. argus*. A similar result has also been obtained in size-dimorphic *C. semilaevis* using molecular functional analysis, in which Wang et al. found that significantly enriched activity in the liver included oxidoreductase activity [2]. However, the energy metabolism gene network underlying SSD is still a relatively poorly explored research area. Our preliminary findings would pave the way for a more complete understanding of this complex regulatory system.

To our best knowledge, energy allocation to gonads is only one aspect of reproduction, some other reproduction-related behaviors, such as finding and competing for mates and reduced feeding, may result in a greater net energy expense and decreased growth [49]. In this study, we also observed that male *S. argus* exhibited significantly higher antioxidant enzymes (SOD, GPX, and CAT) activities compared to females. Similarly, the gender differences in antioxidant defenses have been reported in some other studies. A study of *O. niloticus* showed sex differences in SOD and glutathione S-transferase, with males showing higher activities than female individuals [61]. In brown trout (*Salmo trutta* L.), the liver of females showed higher SOD and CAT activities than that of males [62]. In the zebrafish liver, the expression of antioxidant gene *gpx1* in male liver was significantly higher than in female liver [63]. It is well known that reactive oxygen species (ROS), such as superoxide and hydrogen peroxide, are generated as by-products of aerobic respiration and metabolism, primarily in the mitochondria where energy is generated. Antioxidant enzymes are used to reduce ROS and to limit their harmful effects. Interestingly, endogenous ROS production is associated with diverse physiological processes including changes in the somatic growth of animals [64], and it is hypothesized that oxidative damage might constrain growth rate. Considering that male *S. argus* has more active energy metabolisms compared to females, the higher activity of antioxidant enzymes in the male liver may be correlated with its highly active mitochondrial activities. Our results could support the hypothesis that reduced male growth efficiency is due to increased energy expenditure associated with reproduction [55].

The liver plays a central role in the coordination of various metabolic processes including lipid metabolism. Lipid metabolism is a key process that exerts controlling influences on multiple biological processes such as fish growth, reproduction, and disease-resistance [65]. Dysfunctional lipid metabolism hampers fish growth and reduces the productivity for aquaculture [66]. Recent transcriptomics studies on the liver tissue showed that candidate genes responsible for differential growth rate were overrepresented in lipid metabolism pathway branches [18,25,26,67]. Due to the different metabolic needs for males and females during sexual maturation and reproduction, the fish liver is one of the most sexually dimorphic organs in terms of metabolism. An analysis on the sexual dimorphism of the zebrafish liver demonstrated that males showed activation of transcription factors regulating lipid and glucose metabolisms [63]. A recent investigation on the candidate networks and genes for the SSD of *C. semilaevis* found that certain lipid metabolism-related pathways were significantly enriched [2]. In this study, functional analyses revealed enrichment of lipid transport and lipid binding pathways as well as differential regulation of transcripts involved in fatty acid metabolism. From these enriched pathways, several sex-biased genes such as acyl-coenzyme A thioesterase, carboxylesterase 2, and trans-2-enoyl-CoA reductase were identified (Table S4), some of which have been detected to be differentially expressed between fast- and slow-growing fish strains [24,26], implying that they are potentially linked with differential growth capacity. Additionally, we found that female *S. argus* had higher G6-PDH activity, while male fish exhibited higher HL activity. Higher activity of G6-PDH in female than male may be to satisfy a greater biosynthetic need of female reproduction and breeding [68]. HL is produced exclusively in the liver and it has been reported that estrogen can lower its activity [69]. Taken together, our results could indicate not only an increased lipid synthesis in females but also an enhanced lipolytic response in males, which may both elicit the occurrence of SSD in *S. argus*.

Moreover, sex hormones, particularly estrogens, are demonstrated to drastically shift liver metabolism and have a significant effect on lipid metabolism. In tilapia, E_2_ was demonstrated to induce deposition of lipids by stimulating the expression of genes associated with very low-density lipoprotein assembly and promoting the rate-limiting enzyme in the synthesis of triglyceride [65,66]. Upregulated expression of lipid metabolism-related genes was also observed in the liver of brown trout (*S. trutta* L.) exposed to E_2_ [70]. In yellow perch (*Perca flavescen*), E_2_-downregulated genes represented a variety of functional categories including lipid transport and metabolism [50]. Thus, genes modulating lipid transport and metabolism would be candidates for regulation by E_2_ [50]. In this study, a few genes involved in lipid metabolic processes, such as apolipoprotein E (apoE) and very long-chain acyl-CoA synthetase, were found to be up-regulated in females that exhibit a higher serum level of E_2_ (Table S4). As a component of lipoproteins, apoE is essential to the cellular recognition and internalization of lipoproteins via lipoprotein receptor [71]. In fish species, apoE has been known to be associated with yolk resorption [72]. In agreement with our research, it has been reported that apoE gene is upregulated by E_2_ in perch liver [50]. Collectively, these results suggest an increased uptake of lipoproteins probably stimulated with E_2_ in female *S. argus*, and this result appears reasonable in light of enhanced vitellogenin gene expression in the female liver.

Fish growth is controlled by the somatotropic axis that involves the secretion of pituitary GH and concurrent production of GH-dependent IGF-I by the liver. An increasing number of studies have revealed that the traits of SSD interact with the reproductive axis in fish, and gender-dependent modulation of the GH-IGF axis components is believed to be crucial for the sexual dimorphism of somatic growth [17]. The sexually dimorphic expressions of GH-IGF axis genes have recently been characterized in several fish species that exhibit strong SSD. In general, the faster growth rate of female or male population is attributed to significantly higher levels of *gh* mRNA in pituitary [9,10,11,12] and *igf1* mRNA in liver [12,14,16,73]. In the present study, we found that *igf1* transcript was up-regulated in the liver of female *S. argus*. This result is in agreement with that of our previous research in which *igf1* expression levels were determined to be significantly higher in females than in males at gonadal phase III and IV [16]. The biological actions of IGF-I are further modified by insulin-like growth factor binding proteins (IGFBPs) that act as important players in the IGF system by regulating the half-life of circulating IGF proteins and modulating IGF signaling target tissues. Previous research has demonstrated that IGFBPs can regulate IGF activities depending on the physiological context [74,75]. There is a strong association of low-molecular-weight IGFBPs (e.g., IGFBP-1) with poor growth in teleosts [76]. Here, we observed a higher expression of *igfbp1* gene in the fast-growing females than in the slow-growing males. A similar result has also been reported in *C. semilaevis,* which exhibits female-biased SSD, suggesting regulation of the transport and bioavailability of IGF1 to bind to its receptors at target cells [2]. Given the importance of these genes to teleost growth and the recent reports of estrogen-dependent decrease in *igf1* and *igfbp1* mRNA levels [69,77], further mechanistic study is required to elaborate the involvement of IGF-I and IGFBP-1 in the growth superiority of female *S. argus*.

## 5. Conclusions

The findings in this work showed that the female-biased SSD of *S. argus* could be partially attributed to sexually dimorphic metabolisms in the liver. The higher digestive enzyme activities that could result in a better food utilization efficiency together with increased lipid synthesis may lead to a faster growth rate of female *S. argus*. Also, steroid hormone-mediated sexual dimorphism in energy allocation may be partially related with the formation of SSD. The male liver showed a more active mitochondrial energy metabolism and enhanced lipolytic response, suggesting an increased energy expenditure associated with reproduction, which could be responsible for the reduced growth efficiency of male *S. argus*. Overall, our research provides new insight into the mechanism of differential growth between the sexes, and the results would be of value to further illuminate the nature of SSD in teleost fish.

## Figures and Tables

**Table 1:** Summary of statistics for sequencing data of the liver transcriptomes of *S. argus*.

Sample	Raw Reads (×10^6^)	Raw Bases (Gb)	Clean Reads (×10^6^)	Clean Bases (Gb)	% ≥ Q20	Raito of Clean Read (%)
Female_1	45.53	6.83	45.12	6.77	92.25	99.10
Female_2	44.84	6.73	44.31	6.65	91.51	98.81
Female_3	46.97	7.04	46.49	6.97	92.46	98.98
Male_1	48.40	7.26	47.96	7.19	95.91	99.10
Male_2	45.40	6.81	44.84	6.73	95.90	98.77
Male_3	44.44	6.67	43.92	6.59	96.06	98.84
Mean	45.93	6.89	45.44	6.82	94.01	98.93
Total	275.58	41.34	272.65	40.90	-	-

**Table 2:** Statistical summary of assembly data for the liver transcriptome of *S. argus*.

Category	Value
Unigene number	79,115
Min length (bp)	200
Max length (bp)	17,724
Mean length (bp)	1258
N50 (bp)	2462
N90 (bp)	471
GC (%)	46.86

## Data Availability

The data presented in this study are available on request from the corresponding author. The data are not publicly available due to the agreement with funding bodies.

## Supplementary Materials

The following are available online at https://www.mdpi.com/article/10.3390/life11060589/s1. Table S1. The product numbers of commercial assay kits. Table S2. The primers for RT-qPCR validation. Table S3. The top 20 enriched GO terms in the differential expression genes (DEGs). Table S4. Searching for differential expression genes (DEGs) involved in material and energy metabolism, metabolites and ion transporters, immune system, and signal transduction. Figure S1. Gonadal sections (H&E staining) of male and female *S. argus*. Scale bars represent 50 µm. Figure S2. Liver sections (H&E staining) of male and female *S. argus*. Scale bars represent 50 µm. Figure S3. Sequence size distribution of assembled unigenes derived from the *S. argus* liver transcriptomes. Figure S4. Statistical summary of the annotation analysis of unigenes. (A) Diagram of unigene annotation results against Nr, Swiss-Prot, Nt, KOG and KEGG databases. The number in each colored block indicates the number of unigenes annotated by single or multiple databases. (B) E-value distribution of BLAST search against the Nr database for each unique sequence (E-value 1 × 10^−5^). (C) Identity distribution of BLAST hits for each unique sequence. (D) Species distribution of the BLAST hits for annotated unigenes (E-value 1 × 10^−5^). Figure S5. Gene Ontology (GO) functional classification of annotated unigenes. The unigenes showing significant similarity to homologous genes in GO databases were assorted to three main categories: cellular components, molecular functions, and biological processes. Figure S6. Kyoto Encyclopedia of Genes and Genomes (KEGG) pathways analysis of annotated unigenes. The unigenes were assigned into six main categories: genetic information processing, metabolism, cellular processes, organismal systems, environmental information processing and human diseases. Figure S7. Comparative evaluation of the activity of hepatic enzymes related to carbohydrate metabolism between male and female *S. argus*. Error bars indicate the SEM (*n* = 5). ns: not significant (*p* > 0.05).
