# Supplementary figures and images for: Comparative Physiological and Transcriptomic Profiling Offers Insight into the Sexual Dimorphism of Hepatic Metabolism in Size-Dimorphic Spotted Scat (Scatophagus argus)
Source: Life (Basel). 2021 Jun 21;11(6):589. doi: 10.3390/life11060589 (PMC8233746; doi:10.3390/life11060589)

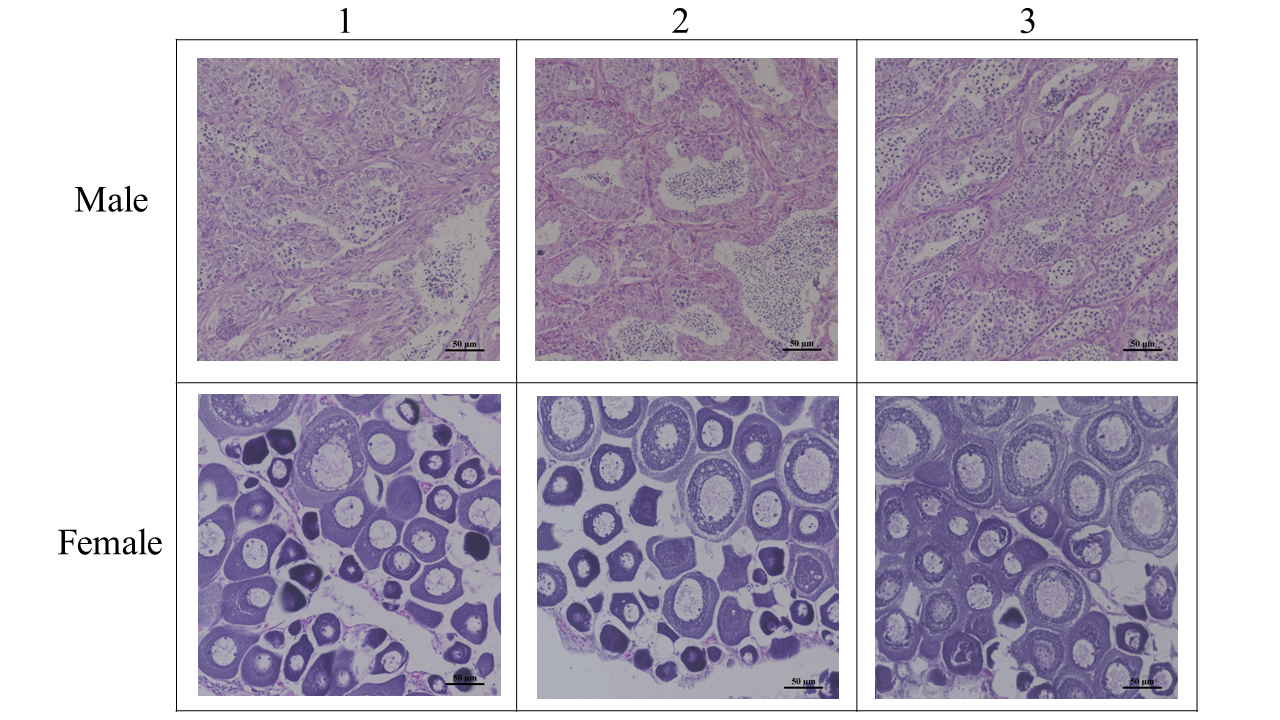

Supplement: Supplementary file 1 [file life-11-00589-s001.zip › Figure S1.tif]

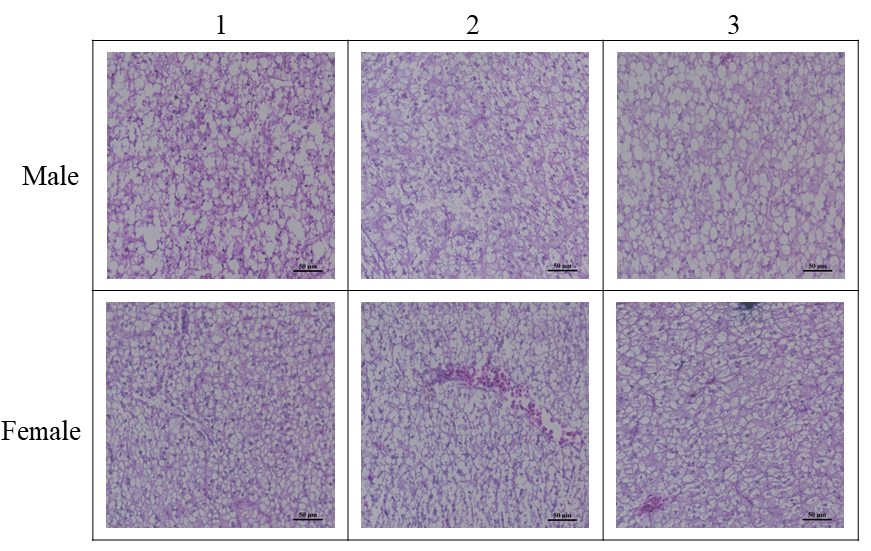

Supplement: Supplementary file 1 [file life-11-00589-s001.zip › Figure S2.tif]

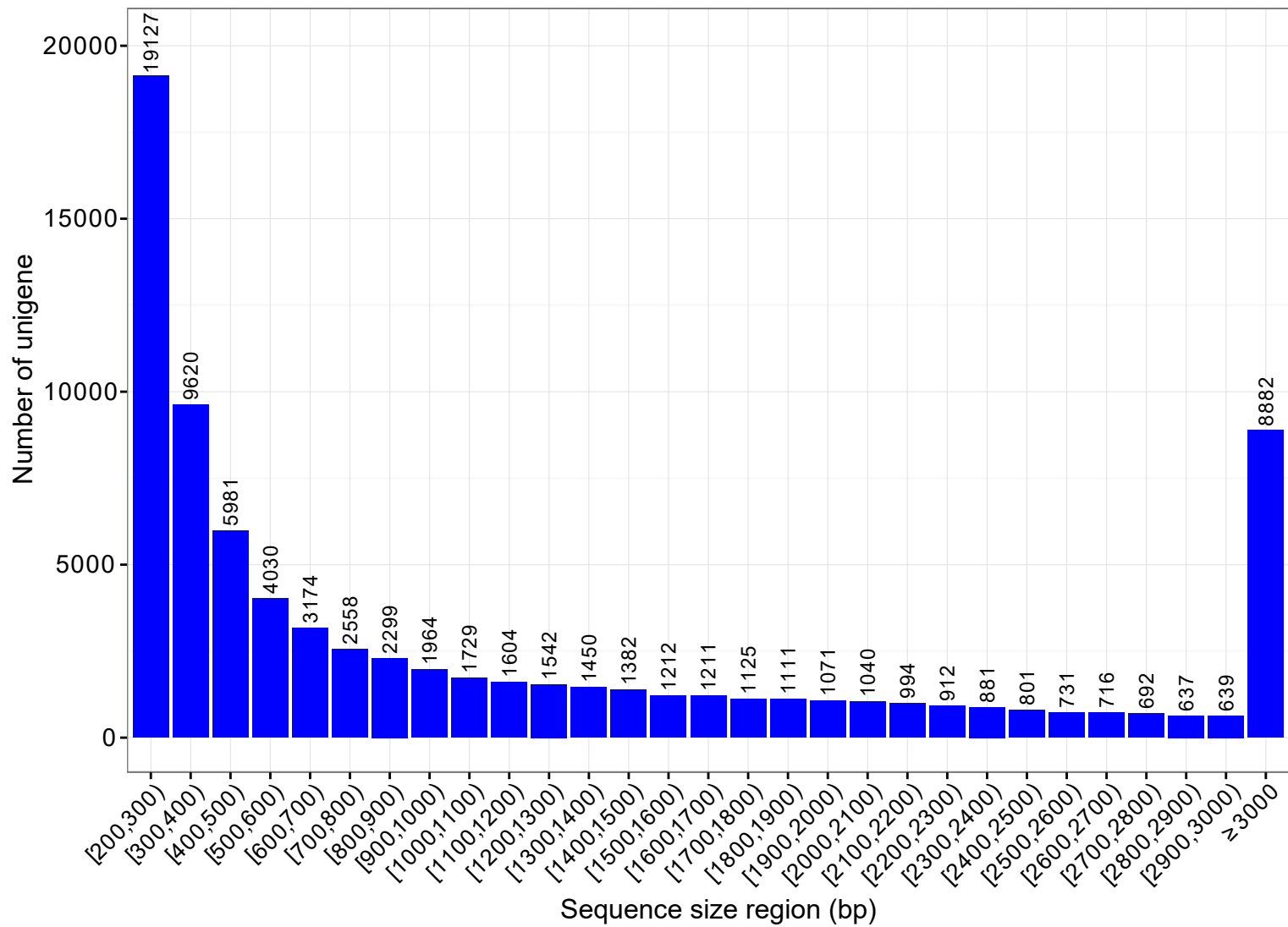

Supplement: Supplementary file 1 [file life-11-00589-s001.zip › Figure S3.pdf]

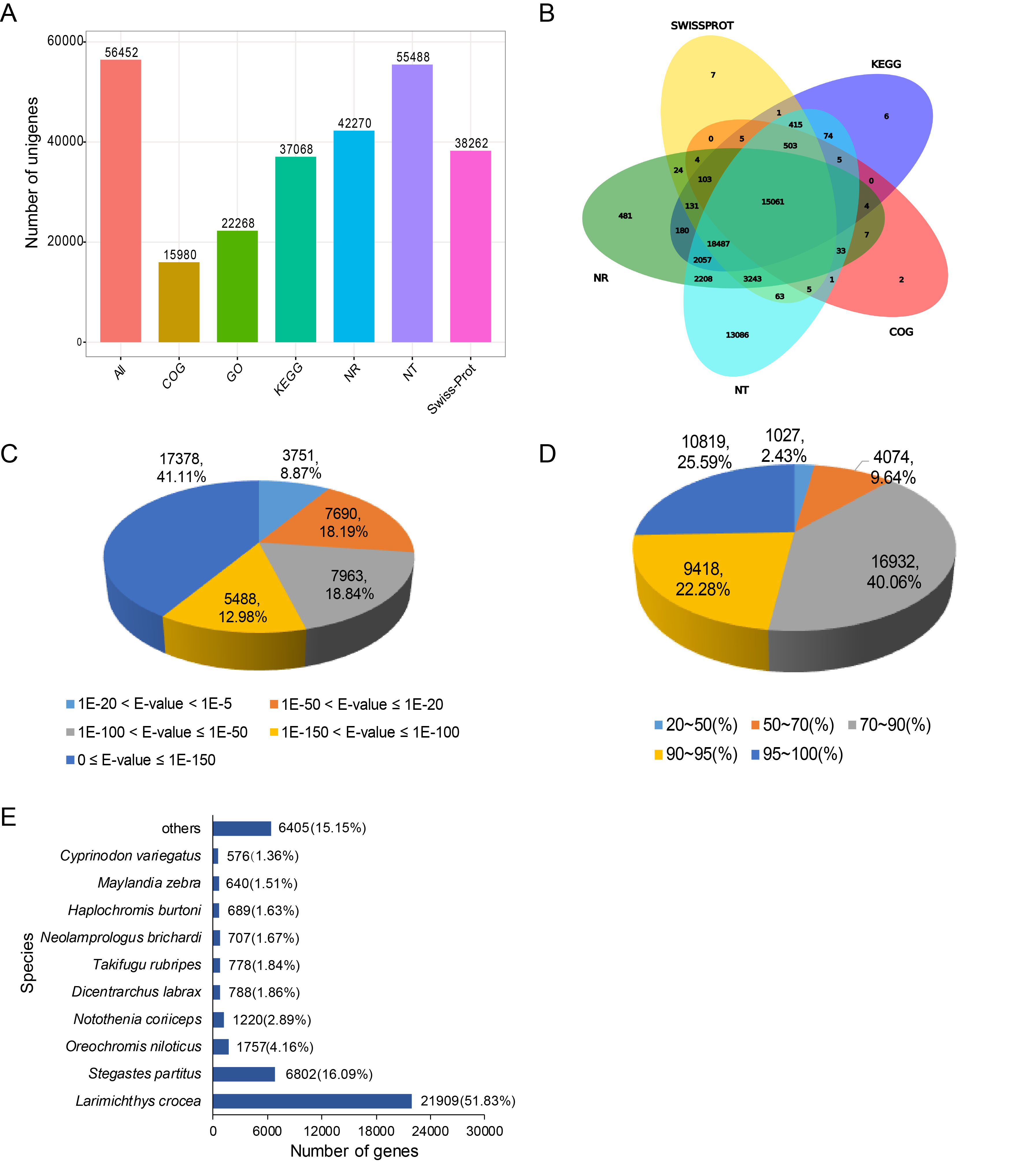

Supplement: Supplementary file 1 [file life-11-00589-s001.zip › Figure S4.tif]

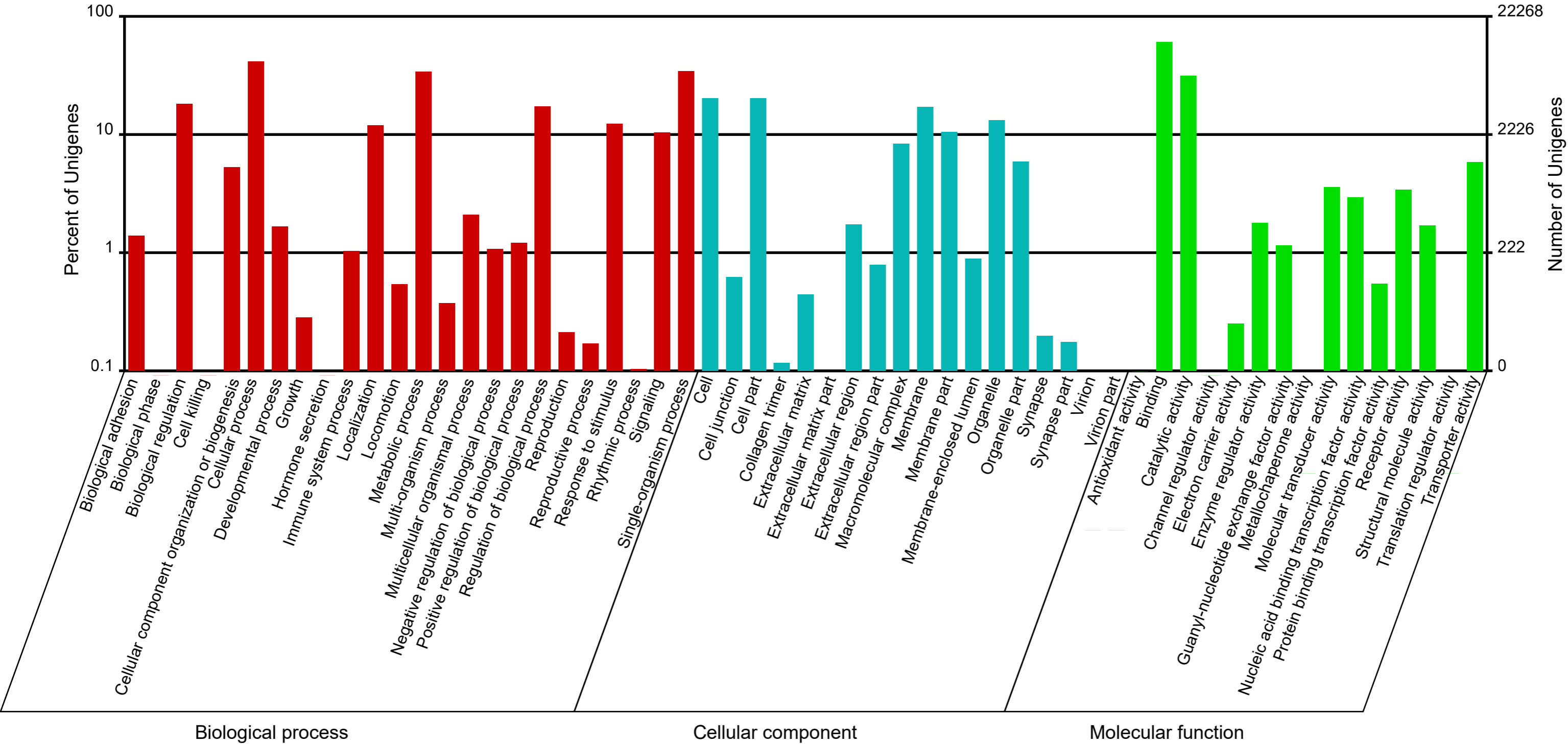

Supplement: Supplementary file 1 [file life-11-00589-s001.zip › Figure S5.pdf]

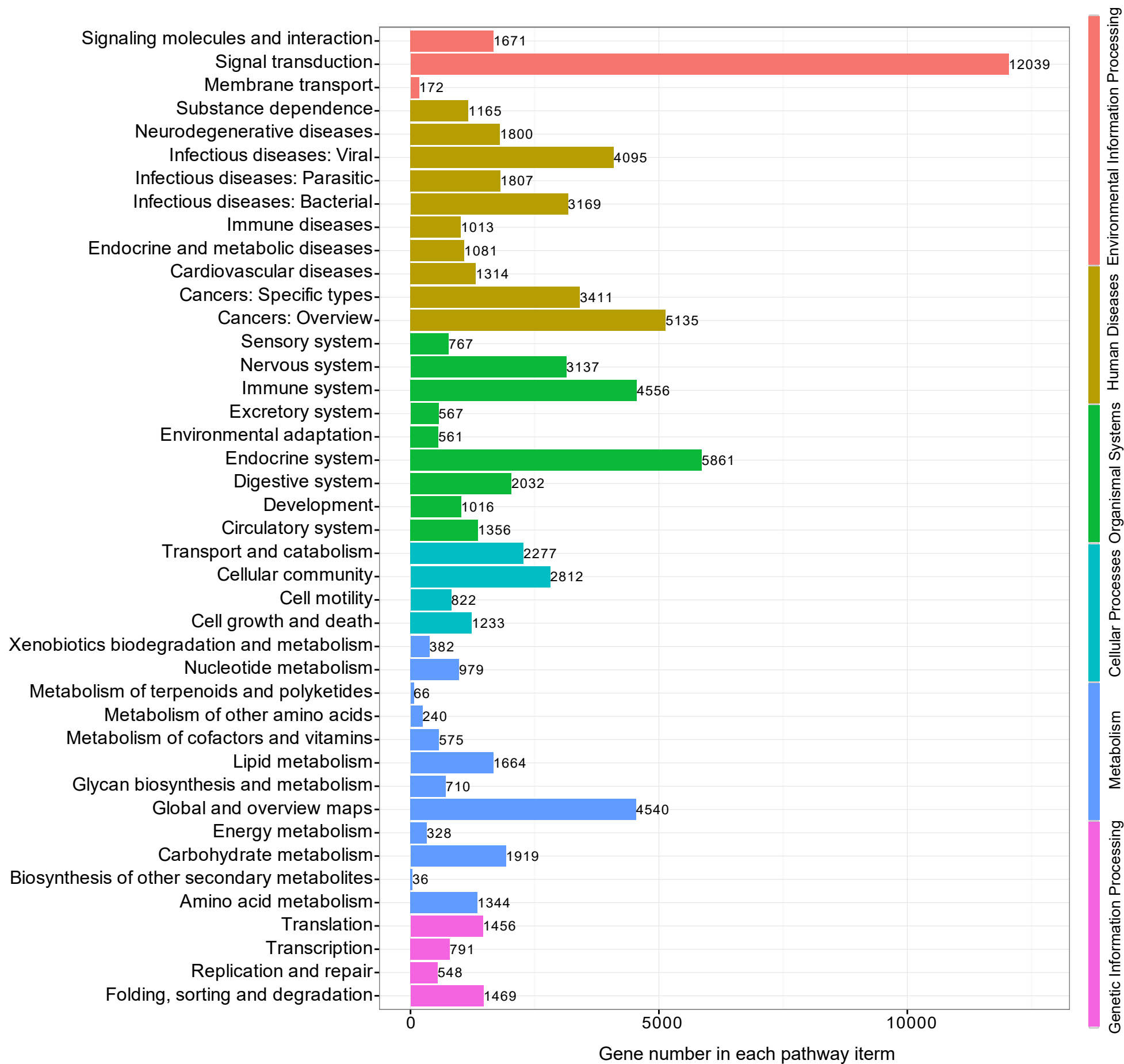

Supplement: Supplementary file 1 [file life-11-00589-s001.zip › Figure S6.pdf]

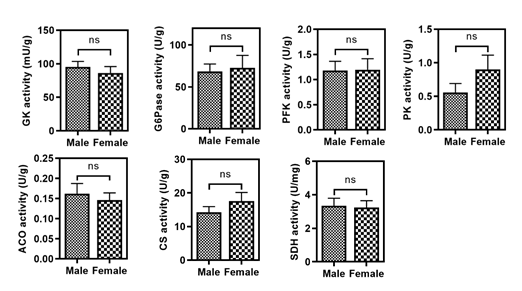

Supplement: Supplementary file 1 [file life-11-00589-s001.zip › Figure S7.tif]
